# Measuring Health Literacy Regarding Infectious Respiratory Diseases: A New Skills-Based Instrument

**DOI:** 10.1371/journal.pone.0064153

**Published:** 2013-05-28

**Authors:** Xinying Sun, Juan Chen, Yuhui Shi, Qingqi Zeng, Nanfang Wei, Ruiqian Xie, Chun Chang, Weijing Du

**Affiliations:** 1 Department of Social Medicine and Health Education, School of Public Health, Peking University, Beijing, China; 2 Chinese Center of Health Education, Beijing, China; Tehran University of Medical Sciences, Islamic Republic of Iran

## Abstract

**Background:**

There is no special instrument to measure skills-based health literacy where it concerns infectious respiratory diseases. This study aimed to explore and evaluate a new skills-based instrument on health literacy regarding respiratory infectious diseases.

**Methods:**

This instrument was designed to measure not only an individual’s reading and numeracy ability, but also their oral communication ability and their ability to use the internet to seek information. Sixteen stimuli materials were selected to enable measurement of the skills, which were sourced from the WHO, China CDC, and Chinese Center of Health Education. The information involved the distribution of epidemics, immunization programs, early symptoms, means of disease prevention, individual’s preventative behavior, use of medications and thermometers, treatment plans and the location of hospitals. Multi-stage stratified cluster sampling was employed to collect participants. Psychometric properties were used to evaluate the reliability and validity of the instrument.

**Results:**

The overall degree of difficulty and discrimination of the instrument were 0.693 and 0.482 respectively. The instrument demonstrated good internal consistency reliability with a Cronbach’s alpha of 0.864. As for validity, six factors were extracted from 30 items, which together explained 47.3% of the instrument’s variance. And based on confirmatory factor analysis, the items were grouped into five subscales representing prose, document, quantitative, oral and internet based information seeking skills (χ^2^ = 9.200, P>0.05, GFI = 0.998, TLI = 0.988, AGFI = 0.992, RMSEA = 0.028).

**Conclusion:**

The new instrument has good reliability and validity, and it could be used to assess the health literacy regarding respiratory infectious disease status of different groups.

## Introduction

In recent years, the viruses SARS, influenza (H5N1 and H1N1) have broken out, and drug-resistant tuberculosis has made a resurgence, attracting public attention to infectious respiratory diseases. The most important feature of these diseases is that they can be prevented, controlled and cured. Correct preventive measures and health behavior can stop their spread to a very large extent. So health education and health promotion is both efficient and important in this field. Low health literacy can restrict and impact on the efficiency of health education, adoption of preventative measures and health behaviors.

After more than 20 years’ development, health literacy has several different definitions. The Ratzan and Parker (2000) definition has been widely used. As stated in *Healthy People 2010,* health literacy was defined as “The degree to which individuals have the capacity to obtain, process, and understand basic health information and services needed to make appropriate health decisions.” [1] Berkman in 2010 gave some suggestions for minor modifications to the definition: “The degree to which individuals can obtain, process, understand, and communicate about health-related information needed to make informed health decisions.” [2].

Different definitions adopt different measurement indicators and assessment systems. The most common measurement and assessment tools include Rapid Estimate of Adult Literacy in Medicine (REALM) [3], Test of Functional Health Literacy in Adults (TOFHLA) [4], and the Department of Education’s 2003 National Assessment of Adult Literacy (NAAL) [5]. REALM was developed as a quick screening tool to assist physicians in identifying patients with limited reading skills, and to help estimate patients’ reading levels. It contains 66 common medical terms and nouns representing body parts and diseases. REALM emphasizes language capability, which reflects people’s ability to recognize terms, but ignoring their understanding and skill in health literacy. TOFHLA measured patients’ numeracy and reading levels in a healthcare environment, and assessed their health literacy. The NAAL scale included information about medicines and prevention and guidance in health care, aimed at assessing subjects’ position and understanding of health-related information and skills, but it did not take into account respondents’ ability to communicate with doctors, or their understanding of medical terms. In 2009, McCormack designed a new skills-based instrument, titled the Health Literacy Skills Instrument (HLSI) to measure health literacy more comprehensively. It measured not only print literacy but also oral and internet-based information seeking skills. [6].

China carried out a National Health Literacy Survey in 2007 to understand Chinese residents’ level of basic health commonsense and skills. [7] A composite index and percentages were adopted to assess health literacy. However, the project mainly indicated residents’ knowledge levels and health-related behaviors, and did not sufficiently reflect their skills.

All these instruments involved all respects of health. Some studies tried to measure health literacy in the field of infectious diseases, but they were knowledge or perception-based. [8], [9] There is no special instrument to measure skills-based health literacy where it concerns infectious respiratory diseases. Given that health literacy assessment in certain fields would be more particular, comprehensive and specific, our research aimed only to assess health literacy as it relates to respiratory infectious diseases.

This study was supported by the China-US Collaborative Program which focused on emerging and re-emerging infectious diseases. Due to the project’s background, this study aimed to develop a skills-based instrument to measure the health literacy concerned only with infectious respiratory diseases. The development of the instrument and its reliability and validity are described in this article.

Compared with other health literacy measurement tools mentioned above, this instrument used audiovisual stimuli to measure oral literacy, and computer and internet questions to measure internet-based information seeking skills, other than print stimuli to measure print literacy, so as to explore the feasibility and reliability of a skills-based measuring method. Additionally, in terms of content domains, it reflects health related issues for disease prevention, health care maintenance and treatment, and the available health system utilization, which can be used to assess people’s health literacy not only in a clinical setting but also in a public environment. This kind of assessment can provide directions or methods of how to conduct patient education and public health education on the treatment and prevention of infectious respiratory diseases.

## Methods

### Instrument Development

The questionnaire is a self-administered test, consisting mainly of single choice questions. It takes 20 to 50 minutes to finish. The average finishing time is 30 minutes. The first part of the questionnaire establishes the social demographic characteristics of the participant. It measures age, gender, ethnicity, household registration status, marital status, education, occupation and income.

The main part of the questionnaire measures and assesses health literacy. We used Ratzan and Parker’s (2000) definition of health literacy: “The degree to which individuals can obtain, process, understand, and communicate about health-related information needed to make informed health decisions”. [1].

We selected appropriate health information to measure according to the study’s objectives using the following selection criteria:

- It covers all prevention and control information about infectious respiratory diseases among the public, without regional and sex differences.
- It covers all transmission vectors for infectious respiratory diseases related to the general public.
- The health information should be objective, scientific, direct and vivid.
- It should not take more than one minute to read each piece of information.

Sixteen stimuli materials (See Table S1) were included in the questionnaire, sourced from the World Health Organization (WHO), the Chinese Center of Disease Control and Prevention and the Chinese Center of Health Education. Information involved the distribution of epidemics, immunization programs, early symptoms, means of disease prevention, preventative behavior for individuals (for example, washing hands, wearing a face mask, sneezing), use of medications and thermometers, treatment plans and the locations of hospitals. The media forms contained brochures, books, posters, newspapers, website information, a hotline number and items including a medicine box, thermometer and hospital map. We categorized tasks into three skills set areas as follows: print, oral and internet information seeking. Moreover, the ‘print’ skill set included three aspects. The print-prose scale measured the knowledge and skills needed to search, comprehend, and use information from texts that were organized in sentences or paragraphs, while the print-document scale measured from non-continuous texts in various formats. And the print-quantitative scale measured the knowledge and skills needed to identify and perform computations using numbers embedded in printed materials. [5].

The questionnaire consisted of 30 items, aimed to assess individuals’ capacity to obtain, interpret and use information. Tasks were classified as follows: understanding health-related text; interpreting information in the form of pictures, symbols, and maps videos and recordings; interpreting information in the form of videos and recordings; completing computations; applying information to a specific scenario; and utilizing the internet to obtain health information. These selective questions had one correct answer, a correct reply was worth one point, while an incorrect reply scored zero. Therefore, the maximum possible score for this part of the questionnaire was 30 points.

### Target Population and Sampling

The author believes that the rising trend of infectious respiratory diseases is probably related to greater population mobility. Due to the accelerating process of urbanization in China, the number of internal migrants in cities is growing. They are living and working in closer confines, with facilities shared between more people. These people aid the outbreak and spread of contagious diseases. Therefore, cities characterized by dense populations and rapid population growth should be the crucial locations for research about health literacy related to infectious respiratory diseases. [10].

Field work in Beijing (The capital of China), Datong (in Shanxi province, North China) and Shenzhen (in Guangdong province, South China) was conducted from May to December 2011. Sample size was calculated by the function *n* = *Z^2^_1-a/2_P* (1 − *P*)/*d* ^2^ × deff. The National Health Literacy Survey in 2008 regarding health literacy towards infectious diseases showed the expected percentage was 16% (*P* = 0.16). [11] The confidence interval was ±10%, *d* = 0.1*P = 0.016, α = 0.05, *Z_1-α/2_* = 1.96, deff is design effect, defined as 2 due to cluster sampling. Therefore the minimum sample size is 2581. If recovery rates and efficiency rates of the questionnaire are to be both 90%, then the actual sample size should be at least 3186. So the sample size in each of the three cities surveyed would have to be about 1062. In the end, 3222 residents responded to the survey.

Multi-stage sampling was employed to collect data from each city. The target population was first stratified into local and non-local residents, with an equal sample size for each group. Then, based on the principle of balancing samples among factors like age and occupation, cluster sampling was conducted in six places where local residents gather (including communities, government organizations, factories and other institutions) and six places where non-local residents gather (including building sites, hotels, employment medical examination centers and assembly shops).

### Ethics Statement

The study protocol was reviewed and approved by the Peking University Institutional Review Board and it was in accordance with Helsinki Declaration. Written informed consent was obtained for all study participants.

### Statistical Analysis

Questionnaires with more than 10% of items unanswered were considered ineligible and removed before analysis in order to ensure the quality of data. EPI Data 3.0 was used for data double entry and SPSS 13.0 for data analysis. Descriptive statistics were employed to examine demographic characteristics. Discrimination index (D) was calculated by the score of the top 27% of participants (P_H_) minus that of the bottom 27% (P_L_). Scale and factor analyses were conducted to verify the scale’s reliability and construct validity. Confirmatory factor analysis of a half randomly selected sample was implemented by the Statistical Analysis System (SAS 6.12). Covariance Analysis of Linear Structural Equations (CALIS) was performed to examine the model. In addition, maximum likelihood estimation was used to appraise the parameters with a covariance matrix. Participants were classified into three categories based on their health literacy levels: proficient, basic, and below basic. Cut-points were determined through receiver operating characteristic (ROC) analyses based on their educational level. Generally, the α level was set at 0.05. Initial eigenvalue >1 was the criterion in the factor analysis.

## Results

### Participant Characteristics

Table 1 shows participants’ mean health literacy scores by characteristics. Health literacy score increased as education levels and income rose, but declined along with age. There was a significant difference in scores across three categories of occupation. The first was between students and scientific and technical workers/teachers/doctors, the second included office workers, service providers, general workers and others, and the third included the retired and farmers.

**Table 1:** Comparison of health literacy scores by participant characteristics.

Participant characteristics	N	%	Health literacy Score				
			Mean	Std.	*SE*	*t/F*	*P*
Gender							
Male	1550	48.7	21.13	5.90	0.15	2.847	0.004
Female	1636	51.3	20.53	6.14	0.15		
Age (years)							
16–22	882	27.8	22.51*	4.85	0.16	119.911	0.000
23–29	684	21.6	22.65*	5.12	0.19		
30–39	610	19.3	21.74*	5.66	0.23		
40–49	502	15.8	19.04^†^	6.28	0.28		
50–59	283	8.9	16.28^‡^	6.46	0.38		
60+	207	6.5	15.61^‡^	5.91	0.41		
Education							
Less than middle school	206	6.5	14.32*	6.12	0.43	311.262	0.000
Middle school graduate	1016	31.9	18.21^†^	5.85	0.18		
High school graduate	1277	40.1	21.93^‡^	5.28	0.15		
More than high school	683	21.5	24.51^#^	4.23	0.16		
Census							
Local registered	1480	46.3	21.24	6.31	0.16	3.775	0.000
Non-local registered	1718	53.7	20.43	5.78	0.14		
Nationality							
Han majority	3090	96.3	20.86	6.03	0.11	2.458	0.014
The minority	119	3.7	19.48	5.66	0.52		
Marital status							
Not married	1291	40.6	22.79*	4.82	0.13	126.336	0.000
Married	1809	57.0	19.51^†^	6.38	0.15		
Widowed/divorced	76	2.4	18.55^†^	6.90	0.79		
Occupation							
Scientific and technical worker/teacher/doctor	298	9.2	24.04*	4.73	0.27		
Student	54	1.7	24.41*	4.61	0.63		
Office worker	130	4.0	21.72^†^	6.26	0.55	61.725	0.000
Others	509	15.8	21.61^†^	5.94	0.26		
Factory worker/miner	979	30.4	21.21^†^	5.88	0.19		
Hotel/restaurant waiter/waitress	853	26.5	20.54^†^	5.48	0.19		
Retired	237	7.4	16.11^‡^	5.86	0.38		
Farmer	162	5.0	15.68^‡^	5.85	0.46		
Personal monthly income(RMB yuan)							
<1000	327		17.36*	6.48	0.36	43.721	0.000
1000–1999	1293		21.20^†^	5.73	0.16		
2000–2999	992		20.82^†^	5.76	0.18		
3000–3999	338		22.58^‡^	5.45	0.30		
>4000	185		22.85^‡^	5.74	0.42		

*Note*: The Student-Newman-Keuls method was used to control the total α level. There were significant differences between *, ^†^,^‡^ and ^#^ groups, but no significant differences within each group.

### Difficulty, Discrimination, Reliability and Validity of the Instrument

**Figure 1:**
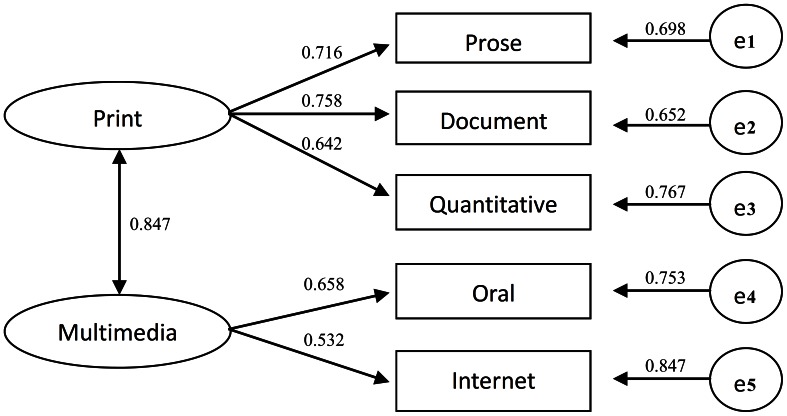
Confirmatory factor analysis of the instrument of health literacy by Covariance Structure Analysis with standard coefficients. Note: “e1” to “e5” were the residuals of variables.

The average percentage of correct answer was 69.3%, that is, the overall degree of difficulty (P) was 0.693. The overall degree of discrimination was 0.482. The instrument had good internal consistency, while the Cronbach’s alpha coefficient was 0.863 (See Table 2). By analyzing these together with the exploratory factors, six factors were extracted from 30 items (the communality of each item is shown in Table 3), which explained 47.3% of total variance. The six factors were named *f_1_*: internet, *f_2_*: oral, *f_3_*: quantitative, *f_4_*: document, *f_5_*: prose and *f_6_*: chart. As *f_3_* and *f_6_* were both about numeracy, and *f_6_* had only two items, they were combined. So the items were regrouped into five subscales representing print-prose, print-document, print-quantitative, oral and internet-based information seeking skills, and each subscale had good internal consistency (See Table 3). The correlation between subscales is shown in Table 4. Based on confirmatory factor analysis (See Figure 1), the items were grouped into five subscales representing prose, document, quantitative, oral and internet based information seeking skills very well (χ2 = 9.200, P>0.05, GFI = 0.998, TLI = 0.988, AGFI = 0.992, RMSEA = 0.028). The latent variable “print” was extracted from “prose”, “document” and “quantitative”, that measured the health literacy to search, comprehend, and use information from print media. The latent variable “multimedia” was extracted from “oral” and “internet”, that measured the health literacy to search, comprehend, and use information from multimedia.

**Table 2:** Psychometric properties of health literacy items regarding infectious respiratory.

Sub-scale^a^/items	% correct (P)	P_H_ b(%)	P_L_ b (%)	D^b^	Item-total correlation	Commu-nality
**Print-prose**						
P1: Which behavior is not helpful for flu prevention?	80.9	97.6	56.9	0.407	0.396	0.429
P2: Which statement is correct about wearing respirators?	67.3	70.7	40.3	0.304	0.376	0.322
P3: Which vaccine is not included in the national essential program of immunization?	70.7	95.2	42.7	0.525	0.414	0.454
P4: Can frequent antibiotic intake prevent catching “supper bacteria”?	66.0	92.5	36.4	0.561	0.404	0.392
P5: For which of the following groups is this medicine relatively safe?	86.1	97.4	70.1	0.273	0.283	0.250
Print-document*						
D1: How many local infected cases of Influenza A (H1N1) appear in Beijing?	51.0	79.9	27.5	0.524	0.332	0.290
D2: What does the OTC mean on the right corner of the pill box?	42.5	66.1	20.9	0.452	0.269	0.207
D3: The thermometer shows the temperature is __ ?	79.9	97.1	52.1	0.450	0.444	0.351
D4: Someone with this body temperature means he has which kind of fever?	74.6	94.6	44.2	0.504	0.441	0.364
D5: In which part of the hospital is the outpatient department located?	80.7	96.9	57.1	0.398	0.374	0.426
D6: Which direction should you choose if you were to go from the outpatient department to the department of internal medicine building?	64.5	90.5	35.1	0.554	0.393	0.479
D7: Which lift and floor should you choose if you want to go to the Ear-Nose-Throat department?	76.4	95.0	54.1	0.409	0.358	0.320
D8: A man has taken treatment for his cold for one week yet not significantly recovered. If he wants to see a professional doctor on Friday morning, whose appointment should he register?	80.0	95.1	58.0	0.371	0.369	0.386
**Print-quantitative**						
Q1: If a man had a fever and he took a pill at 8∶00 am, when should he take a next pill?	76.1	93.4	51.5	0.419	0.377	0.374
Q2: If a man took a pill at 6∶00 am, 11∶00 am, 3∶00 pm and 7∶00 pm, when could he take this medicine once more?	43.6	71.0	22.1	0.489	0.305	0.284
Q3: A man is in his county’s hospital for his asthma. Besides the self-paid part, the total expense is 2200 Yuan. For how much money can he submit an expense account to NCMS office?	61.0	85.6	36.8	0.488	0.322	0.353
Q4: A man is sick and needs to be hospitalized. If the total expense is fixed, in which hospital he will spend least after submitting an expense account to NCMS office?	65.8	87.9	43.7	0.442	0.299	0.362
Q5: What was TB’s ranking in incidence rate?	54.7	83.7	33.8	0.499	0.301	0.705
Q6: Which disease ranked second in fatality rate?	54.2	83.2	34.4	0.488	0.280	0.744
Oral*						
O1: If you want to get information about immunization against measles, which number should you press?	74.1	94.5	48.9	0.456	0.386	0.418
O2: If you want personal consulting, which number should you press?	81.9	98.2	55.4	0.428	0.431	0.453
O3: Which of the following statements is NOT correct? c	71.8	91.3	49.1	0.422	0.304	0.361
O4: Where can you get free diagnosis and treatment for TB according to public policy?	70.7	89.2	51.4	0.378	0.257	0.269
O5: Of the family members in the video, who faced the wrong way when coughing?	71.9	89.8	51.4	0.384	0.292	0.412
O6: Which of the following statements is NOT correct? d	77.7	95.6	51.6	0.440	0.395	0.389
Internet*						
I1: Can you use a computer?	76.7	99.9	39.1	0.608	0.579	0.805
I2: Can you use the internet to acquire health information?	70.9	99.8	29.8	0.700	0.601	0.945
I3: Which search engine do you usually use?	69.8	96.6	30.8	0.658	0.553	0.893
I4: Which Chinese character input system do you usually use?	71.2	99.3	31.4	0.679	0.579	0.933
I5: Can you handle the following task: search for some information about “measles vaccine immunization” using the internet?	65.6	97.8	21.8	0.760	0.606	0.813
**Overall**	69.3			0.482	0.864	

Note: ^a^:The print-prose literacy scale measured the knowledge and skills needed to search, comprehend, and use information from texts that were organized in sentences or paragraphs.

The print-document literacy scale measured the knowledge and skills needed to search, comprehend, and use information from non-continuous texts in various formats.

The print-quantitative scale measured the knowledge and skills needed to identify and perform computations using numbers embedded in printed materials. [5].

The oral literacy scale measured the skills to comprehend and interpret information in the form of videos and recordings.

The internet literacy scale measured the skills to utilize the internet to obtain health information.

b , P_H_ is the score for the top 27%, and P_L_ is the score for the bottom 27%; D = P_H–_P_L._

c , the video describes early symptoms of tuberculosis and some policies about it.

d , the video describes knowledge and skills in daily life to prevent H1N1 flu.

**Table 3:** Reliability and construct validity of the instrument on health literacy regarding infectious respiratory diseases.

Factors	Items	Cronbach α		Eigenvalues	% of Variance
*f_1_*:internet	5(I1,I2,I3,I4,I5)	0.964		6.562	21.874
*f_2_*:oral	6(O1,O2,O3,O4,O5,O6)	0.624		2.779	9.265
*f_3_*:quantitative	4(Q1,Q2,Q3,Q4)	0.530^a^		6.562	4.649
*f_4_*:document	8(D1,D2,D3,D4,D5,D6,D7,D8)	0.664		1.339	4.464
*f_5_*:prose	5(P1,P2,P3,P4,P5)	0.567		1.098	3.659
*f_6_*:chart	2(Q5,Q6)			1.007	3.357
*Total*	30	0.864			47.268

Note: ^a^, it was calculated by six items from *f_3_* and *f_6_*.

**Table 4:** Correlation coefficients of sub-scales of health literacy.

Sub-scale^a^	Overall	Print-prose	Print-document	Print-quantitative	Oral	Internet
Overall	1.000					
Print-prose	0.721**	1.000				
Print-document	0.791**	0.531**	1.000			
Print-quantitative	0.691**	0.468**	0.497**	1.000		
Oral	0.686**	0.413**	0.422**	0.339**	1.000	
Internet	0.690**	0.327**	0.358**	0.256**	0.350**	1.000

Note: **P<0.01.

a The print-prose literacy scale measured the knowledge and skills needed to search, comprehend, and use information from texts that were organized in sentences or paragraphs.

The print-document literacy scale measured the knowledge and skills needed to search, comprehend, and use information from non-continuous texts in various formats.

The print-quantitative scale measured the knowledge and skills needed to identify and perform computations using numbers embedded in printed materials. [5].

The oral literacy scale measured the skills to comprehend and interpret information in the form of videos and recordings.

The internet literacy scale measured the skills to utilize the internet to obtain health information.

### ROC Analysis

An ROC curve was used to analyze the classification of health literacy levels. Comparisons by education level indicated a cut-point of 24 for differentiating respondents with college education versus no college education (sensitivity = 0.64, specificity = 0.71), and a cut-point of 21 for differentiating respondents with an education of at least high school versus less than high school (sensitivity = 0.70, specificity = 0.69). Based on these analyses, we classified participants into three groups: proficient literacy (score >24), basic literacy (score of 21–24), and below basic literacy (score <21). In the sample, 33.5% of participants were found to have proficient literacy, 24.5% basic literacy, and 42.0% below basic literacy.

### Simplified Measurement Tool

Based on these results, a simplified version of the infectious respiratory disease health literacy measurement tool has been developed. One representative item from the prose (P3), quantitative (Q3), oral (O3) and internet (I5) category, and two items from the document (D4, D6) category were selected. The particular items selected were all answered with a moderate accuracy rate (range: 61.0%–74.6%, average: 68.0%) with a good discrimination (range of D: 0.488–0.760, average: 0.542). The items’ importance and practical value for the prevention of infectious respiratory diseases was also taken into account. Therefore, the simplified measurement tool had six items from six stimuli. The Cronbach’s alpha coefficient was 0.571. The simplified version with six items significantly correlated with the full version (r = 0.843, *P*<0.001).

## Discussion

The methods of measuring health literacy have improved with the development of its definition. The earliest prevalent measuring tool of health literacy was the REALM [3], which merely measured adult patients’ ability to read and spell common medical terms, expressing the name of body parts and diseases. As it is not able to comprehensively evaluate patients’ abilities, this measuring tool is no longer fundamental to interpretations of health literacy. Likewise, the 2008 investigation into citizens’ health literacy in China has similar limitations. It tested a wide range of basic health knowledge and skills, but did not measure subjects’ ability to learn and apply new skills [11]. Another commonly used measuring tool named TOFHLA was the first way of measuring functional health literacy. It measures subjects’ efficiency in learning, thinking and processing health information, and evaluates their ability in both counting and reading comprehension. RTI International has explored a measurement tool for health literacy based on skills [6], which expands the measurement to three dimensions, namely print, oral and network information seeking. Interpreting health literacy reasonably and comprehensively, this measuring tool has been chosen as the initial chief source of this study design.

As a measurement tool for functional health literacy, this study’s primary feature is a questionnaire. The answers to all questions, included in corresponding health information materials, can only be obtained after the information is comprehended and processed. This is an implementation of the concept that health literacy is the individual’s ability in obtaining, comprehending and processing basic health information or services, and making right decisions related to health. Using this tool, subjects’ health literacy level will not depend on their previous health knowledge, but rather, on their ability to interpret and understand health information on the spot.

Due to the background of the project’s fund, the information materials used to measure health literacy in this study mainly come from the field of respiratory infectious disease prevention and treatment, causing field limitations in measured health literacy, but increasing the value of information on disease control in this specific field. Comprehensive measurement of health literacy can be achieved by continuing this study while expanding the range of health information materials to other health-related fields.

The instrument has moderate degree of difficulty, good discrimination, good reliability and validity. Using this instrument, we supposed that 58% of Chinese adults had basic literacy on respiratory infectious diseases or better. This is much higher than the data (16%) obtained from the National Health Literacy Survey in 2008 regarding health literacy towards infectious diseases [11]. However, the two studies tested very different aspects of health literacy, using different methods and evaluation criteria.

In accordance with the underlying theory of RTI, this study categorizes health information into five classes, namely reading, picture comprehension, listening, computing and network information searching. This way it can imitate all kinds of information media accessible in daily life, and evaluate subjects’ multi-aspect ability to process information. In this research, the difference in accuracy rates between reading and other skills indicates that the measurement result of reading skills alone cannot completely and objectively reflect subjects’ ability to process all kinds of health information in real life. This substantiates the necessity to measure health literacy from many dimensions. The health information materials used in this study are all officially circulated promotional materials.

Very little time is available for the measurement of health literacy in the health promotion and medical services environment, but health literacy measurement in across some districts and populations is still necessary. Therefore, simplifying the health literacy measurement tool will be necessary in the future. Currently, the simplified versions of many universal measurement tools have been developed so as to adapt for their practical requirements. Taking S-TOFHLA for example, the 17 calculation problems and three reading comprehension questions in the original version have been reduced to four calculation problems and two reading comprehension questions. This has served to enhance the tool’s practical applicability by shortening the test’s duration from around 22 minutes to around 12 minutes [12]. In this study, 30 items were reduced to only six items. The duration of the test was shortened from 20–50 minutes to four-to-10 minutes. Subjects’ decreased tolerance for taking long tests may seriously influence the quality of measurement; therefore, the simplified version may yield more believable results other than the advantage of an expanded applicable scope (conducting quick assessment of patients’ literacy in a medical environment).

Because the sampling method was not strictly randomized, the sample, whose demographic characteristics may be different from that of the total population, cannot perfectly represent the health literacy of all citizens all in mainland China. Limited by the project’s background, this study only measures health literacy where it concerns infectious respiratory diseases. A comprehensive measurement concerning other diseases still needs to be developed. In the pilot survey, wireless internet was used to test internet-based information seeking skills. Due to the low speed of wireless internet, the survey took 10–15 minutes longer to complete than otherwise expected. This affected participants’ enthusiasm for completing the test. Therefore, in the formal study, we used a series of questions to test the ability of internet-based information seeking. In future study, if possible, the test should be conducted online, by then the problem will be solved naturally.

## Supporting Information

Table S1 **Health literacy skill area by task and by health domain.**
(DOC)
