# Supplementary material for: Measuring Health Literacy Regarding Infectious Respiratory Diseases: A New Skills-Based Instrument
Source: PLoS One. 2013 May 28;8(5):e64153. doi: 10.1371/journal.pone.0064153 (PMC3665814; doi:10.1371/journal.pone.0064153)
Supplement: Table S1 — Health literacy skill area by task and by health domain. (DOC) [file pone.0064153.s001.doc]

Appendix: Health literacy skill area by task and by health domain

| Task (number of items) | Contents of stimuli | Skills and stimuli format | | |
| --- | --- | --- | --- | --- |
|  |  | Print (prose, document, or quantitative) | Oral (listening or audiovisual) | Internet-based information seeking |
| Understanding health-related text (5) | A poster describes flu-preventive behaviors. | P1: Which behavior is not helpful for flu prevention? |  |  |
|  | A text describes how to use respirators correctly. | P2: Which statement is correct about wearing respirators? |  |  |
|  | A poster describes the national essential program in China. | P3: Which vaccine is not included in the national essential program of immunization? |  |  |
|  | An article selected from newspaper explaining the association between antibiotic intake and “supper bacteria”. | P4: Can frequent antibiotic intake prevent catching “supper bacteria”? |  |  |
|  | The instruction of acetaminophen, which includes its indications,  dosage, adverse reactions and matters needing attention. | P5: To which of the following groups this medicine is relatively safe? |  |  |
| Interpreting information in the form of pictures, symbols, and maps (4) | A map describes the number of local cases of Influenza A (H1N1), as well as the national total, for different provinces all over China at 11th June 2009. | D1: How many local infected cases of Influenza A (H1N1) appear in Beijing? |  |  |
|  | A picture tells people the meaning of different colors of OTC labels mean. | D2: What does the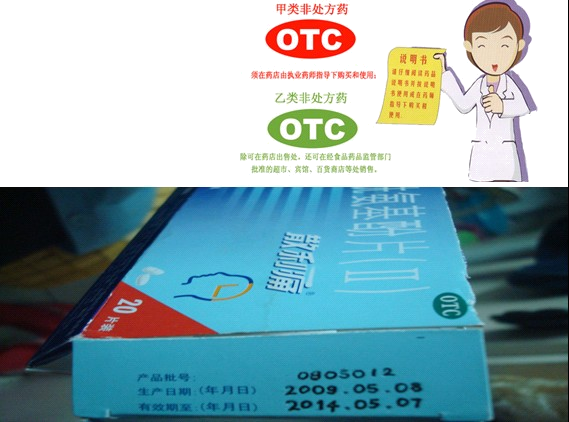 mean on the right corner of the pill box? |  |  |
|  | This picture tells normal range of body temperature, and presents a thermometer. | D3:The thermometer shows the temperature is __ ℃ |  |  |
|  |  | D4: Someone with this body temperature has (which kind of fever?) |  |  |
| Interpreting information in the form of videos and recordings (6) | The record is an automated telephone prompter, guiding the participant in which number to press for different needs. |  | O1: If you want to get information about immunization against measles, which number should you press? |  |
|  |  |  | O2: If you want personal consulting, which number should you press? |  |
|  | The video describes early symptoms of tuberculosis and some policies concerning the disease. |  | O3: Which of the following statements is NOT correct? |  |
|  |  |  | O4: Where can you get free diagnosis and treatment for TB according to public policy? |  |
|  | The video describes knowledge and skills in daily life to prevent H1N1 flu. |  | O5: Of the family members in the video, who did the wrong thing when coughing? |  |
|  |  |  | O6: Which of the following statements is NOT correct? |  |
| Completing computations (6) | Instructions for using acetaminophen, including its symptoms, dosage, adverse reactions and matters needing attention | Q1: If a man had a fever, he took a pill at 8:00 am, when should he next take a pill? |  |  |
|  |  | Q2: If a man took a pill at 6:00 am, 11:00 am, 3:00 pm and 7:00 pm, when could he take this medicine once more? |  |  |
|  | A table describes the expense account submitting system of the New Rural Cooperative Medical System in China | Q3: A man is in his county’s hospital for his asthma. Besides the self-paid part, the total expense is 2200 Yuan. How much money can he submit an expense account to NCMS office for? |  |  |
|  |  | Q4: A man is sick and needs to be hospitalized. If the total expense is fixed, in which hospital will he spend the least after submitting an expense account to the NCMS office? |  |  |
|  | A chart lists the incident rate and case fatality rate of national statutory contagious diseases in China in 2009 | Q5: What was TB’s rank in terms of incidence rate? |  |  |
|  |  | Q6: Which disease ranked second in fatality rate? |  |  |
| Applying information to a specific scenario (4) | A plan sketch of a hospital. | D5: Where is the outpatient department located in the hospital? |  |  |
|  |  | D6: In which direction should you choose to go if you want to go from the outpatient department to the department of internal medicine building? |  |  |
|  | A navigational chart outside the elevators, indicating which lift and floor to choose to different departments. | D7: Which lift and floor should you choose if you aim for [Ear-Nose-Throat](app:ds:ear-nose-throat department) department? |  |  |
|  | A schedule of professional doctors and general doctors, listing their expertise and available time. | D8: A man has taken treatment for his cold for one week yet not significantly recovered. If he wants to see a professional doctor on Friday morning, whose appointment should he register? |  |  |
| Utilizing the internet to obtain health information (5) | Five questions about computer and internet use. |  |  | I1: Can you use a computer? |
|  |  |  |  | I2: Can you use the internet to acquire health information? |
|  |  |  |  | I3: Which search engine do you usually use? |
|  |  |  |  | I4: Which Chinese character input system do you usually use? |
|  |  |  |  | I5: Can you handle the following task: search for some info about “measles vaccine immunization” using the internet? |
